# Consideration in the management of renal cell carcinoma during the COVID-19 Pandemic

**DOI:** 10.1590/S1677-5538.IBJU.2020.S108

**Published:** 2020-07-27

**Authors:** Stênio de Cássio Zequi, Diego Abreu

**Affiliations:** 1 Fundação A. Prudente Divisão de Urologia A.C. Camargo Cancer Center São Paulo Brasil Divisão de Urologia, A.C. Camargo Cancer Center, Fundação A. Prudente, São Paulo, Brasil; 2 Instituto Nacional de Ciência e Tecnologia em Oncogenômica e Inovação Terapêutica – INCIT – INOTE Fundação A. Prudente A. C. Camargo Cancer Center São Paulo Brasil Instituto Nacional de Ciência e Tecnologia em Oncogenômica e Inovação Terapêutica – INCIT – INOTE, A. C. Camargo Cancer Center, Fundação A. Prudente, São Paulo, Brasil; 3 Latin American Renal Cancer Group – LARCG São PauloSP Brasil Latin American Renal Cancer Group – LARCG, São Paulo, SP, Brasil; 4 Pasteur Hospital Department of Urology Montevideo Uruguay Department of Urology, Pasteur Hospital, Montevideo, Uruguay

**Keywords:** Carcinoma, Renal Cell, Nephrectomy, Prostatic Hyperplasia, COVID-19 [Supplementary Concept], Pandemics

## Abstract

**Introduction:**

Recently the COVID-19 pandemic became the main global priority; main efforts and health infrastructures have been prioritized in favor of COVID-19 battle and the treatment of benign diseases has been postponed. Renal cell cancer (RCC) patients configure a heterogenous populations: some of them present indolent cases which can safely have postponed their treatments, others present aggressive tumors, deserving immediate care. These scenarios must be properly identified before a tailored therapeutic choice.

**Objectives:**

We propose a risk- based approach for patients with RCC, to be used during this unprecedented viral infection time.

**Materials and Methods:**

After a literature review focused in COVID-19 and current RCC treatments, we suggest therapeutic strategies of RCC in two sections: surgical approach and systemic therapy, in all stages of this malignance.

**Results:**

Patients with cT1a tumors (and complex cysts, Bosniak III/IV), must be put under active surveillance and delayed intervention. cT1b-T2a/b cases must be managed by partial or radical nephrectomy, some selected T1b-T2a (≤7cm) cases can have the surgery postponed by 60-90 days). Locally advanced tumors (≥cT3 and or N+) must be promptly resected. As possible, minimally invasive surgery and early hospital discharge are encouraged. Upfront cytoreduction, is not recommendable for low risk oligometastatic patients, which must start systemic treatment or even could be put under surveillance and delayed therapy. Intermediate and poor risk metastatic patients must start target therapy and/or immunotherapy (few good responders intermediate cases can have postponed cytoreduction). The recommendation about hereditary RCC syndromes are lacking, thus we recommend its usual care. Local or loco regional recurrence must have individualized approaches. For all cases, we suggest the application of a specific informed consent and a shared therapeutic choice.

**Conclusion:**

In the pandemic COVID -19 times, a tailored risk-based approach must be used for a safe management of RCC, aiming to not compromise the oncological outcomes of the patients.

## INTRODUCTION

Renal cell carcinoma (RCC) is one of most lethal urologic tumors, accounting for 2-3% of all adult malignances. In the 2018 there were 415,000 new cases and 180,000 deaths by this RCC in the World. In the global scenario, the incidence, mortality and prevalence of RCC in Latin America and Caribe corresponds respectively to 7,9% (31,983 cases); 8.2% (14,288) and 7,6% (77682 cases) of World total rates ( 1 ) RCC incidence is increasing, and its main risks factors are competing with the higher risks groups for COVID 19 infection and complications: age >60 years, arterial hypertension, diabetes, obesity, smoking ( 2 ). Thus, during this pandemic time, many patients diagnosed with RCC, if immediately treated by inpatient procedures (as surgery) are under risk of developing this viral infection and its life-threatening complications ( 3 ).

Based on reports from first countries affected by this infection, health authorities, and medical societies, in these times, the main efforts and health infrastructures must be prioritized in favor of COVID-19 battle, reserving in advance, hospital health care facilities, personal protection equipment, and human resources that must be dedicated for pandemic cases. Concomitantly, surgeries for benign affections has been postponed. Regarding oncological patients (in this text we are focused only in RCC) there are challenging tasks: we must develop individualized risk-based therapeutic strategies aiming do not compromise the oncologic outcomes of the distinct risk groups of RCC patients. We must identify cases of RCC with reduced potential of biologic aggressiveness. These patients can be spared from infectious risks associated with immediate surgeries, and we must postpone their treatments. Conversely, it is essential to indicate prompt surgical or systemic treatment for patients presenting with advanced life-threatening tumors despite of actual virus risks.

For patients by personal reasons that are not able to postpone their treatments, individualized shared decision between them, their relatives and physicians must be done after an extensive discussion evolving risks and benefits.

Based on recent information about oncological management of cancer in the new coronavirus era, and the natural history of RCC and best practices of its treatment, we proposed a risk-adapted approach of patients with RCC.

In all mentioned situations, we recommend the signature of a specific informed consent document focused on the adapted risks of RCC managing in COVID-19 times.

All recommendations in this manuscript are for patients not infected by COVID 19. For patients infected, or under suspicion of this infection, and requiring prompt oncologic treatments, we must wait the recovery of the infection to start surgery or systemic approach. Emergency patients must be operated following strict protection recommendations.

## MATERIALS AND METHODS

We reviewed the recent literature (until April 2020 30Th) in English, Spanish and Portuguese Languages, searching by the mesh terms: COVID-19, coronavirus and renal cell carcinoma, kidney cancer, renal cancer, surgery, nephrectomy, ablation, active surveillance, systemic therapy, immunotherapy, target therapy, adjuvant, neoadjuvant.

We discuss the therapy of RCC in two sections below: surgical approach, systemic therapy.

## RESULTS

### Surgical Approach

Surgery (radical nephrectomy, partial nephrectomy) is the most effective treatment for localized and locally advanced tumors and for some selected metastatic cases. Here, we discuss alternatives for small renal masses (SRM), localized tumors, local advanced tumors, and metastatic patients ( 4 ).

### Small Renal masses (tumors ≤ 4.0 cm, and complex renal mass Bosniak II/IV) (cT1aN0M0)

SRM configures a heterogeneous group of lesions with distinct aggressiveness: around 20% correspond to benign lesions, around 60% are RCC with low malignance potential (low grade and favorable histopathologic features), and stage cT1N-0MO staging. These patients, and patients with Complex Cysts (Bosniak III/IV) must be put under active surveillance (AS) with a cross sectional image exam being required in 6-8 months. After the pandemic control they must be treated. For elderly or sick patients, the watchful waiting (WW) is the best approach, avoiding image or laboratory tests. Few SRM (10-20%) can be higher risk tumors (high grade, necrose, aggressive histology). Regarding of these factors, the majority of malignant SRM grows at a rate of few mm/year, and in face of these, it seems safe to maintain these patients under AS, during the estimated few months of the viremia peak and avoid renal biopsy in this moment ( 5 , 6 ).

Exceptions: as some of SRM patients may be refractory, or due to personal reasons are not available to be under AS protocols, we can offer outpatient percutaneous ablation (cryotherapy or radiofrequency), which are as effective as partial nephrectomy (PN), for lesions ≤ 2.5-3.0. For lesions between 3-4.0 cm, PN present best oncological results and can be offered, preferentially by minimally invasive PN (videolaparoscopy, robotic PN, or open PN by mini-incisions ( 7 )), aiming prompt hospital discharge (24h).

### Localized RCC (cT1b:4.0-7.0cm and cT2 N0M0)

Patients presenting cT1b (and some selected T2a< 7.0 cm) lesions without clinic and radiologic evidence of aggressive disease may be safely have postponed their surgeries for around 60-90 days (an image exam (TC or MRI) may be desirable after this time). In this conditions elderly and sick people, must be put under WW. Few young and health patients and patients afraid of postponement, exceptionally, could be ordered an outpatient percutaneous renal mass biopsy: If they present low aggressive lesions (low grade clear cell RCC, Papillary Type 1, chromophobe RCC e.g.), they might be recommended to delay the surgery. If biopsy reveals aggressive patterns or patient is refractory, surgery can be offered. Majority of patients with cT2 lesions must undergo immediate surgery. If they are unfit to surgery, or present co-morbidities which put them under higher risk of COVID-19 infection and complications, we can discuss WW or exceptionally a decision based on their biopsy finding (not for unfit, only for refractory patients). In these cases, the choice between PN or radical nephrectomy is a surgeon's decision-making process, based on his personal skills, the tumor morphometry, patient health status, institutional infrastructure resources, patient preferences, etc. In all cases it will be recommended whenever possible minimally safe invasive approaches (protection against aerosols) and early hospital discharge.

### Locally advanced disease or metastatic lesions (T3-4, and/or N+M0)

Patients with localized advanced RCC, presenting invasion of perirenal fat, renal sinus, excretory system, regional lymph nodes, renal vein, vena cava thrombus or adjacent invasions demand immediate surgical resection. The majority of these require radical nephrectomy (RN), with regional lymphadenectomy and/or wide regional excision. Few SRM (10-15%) can be invasive (cT3) ( 8 ) demanding prompt resection. Efforts for early hospital discharge must be pursued.

### Metastatic cases

Patients presenting poor risk or intermediate risk metastatic RCC according to IMDC- International Metastatic Disease Consortium or MSKCC – Memorial Sloan Kettering classifications - seem to have no advantage with upfront cytoreduction ( 9 , 10 ) and they must receive systemic treatment, which will be discussed in the next section. Although low risk oligometastatic patients can be satisfactory undergo upfront surgery with or without resection of metastasis, we think during the coronavirus time, the upfront surgery must be avoided. Low risk and selected intermediated risk patients can start systemic therapy and must be reevaluated after 16 weeks, the good responders can benefit by the delayed surgical intervention. ( 10 ) Very selected cases of low risk oligometastatic patients, can be put under AS, as in Rini's series ( 11 ), in which the median time free of systemic treatment was around 14,9 months, with no prejudice in disease progression or deaths. Patients with solitary metastases, in our opinion can have its resection postponed or undergo systemic therapy. Palliative nephrectomy for very symptomatic patients (e.g. uncontrollable pain, hypertension, hematuria) may rarely be necessary.

### Local recurrences

Surgical resection is the better treatment of local recurrences. During SARS-CoV-2 pandemic, we think asymptomatic, small and insidious recurrent lesions can be managed by surveillance until the pandemic end. Symptomatic cases with local complications must be prompted resected. Systemic therapy can be individually discussed, also.

### Hereditary RCC

There is no literature regarding hereditary RCC syndromes and COVID-19. We think that majority of these cases can be usually managed, only treating renal lesions ≥ 3.0cm. An exception is the hereditary leiomyomatosis and renal cell carcinoma (HLRCC) an aggressive disease for which immediate surgery is always recommended and systemic drugs are not available and ablatios has not proved efficient ( 12 ).

### Systemic therapy

The scenario involving RCC patients and the indication of systemic treatment in the course of the COVID-19 pandemic, raises a series of questions that are the focus this discussion. The two main questions that arise are, in the first place, whether patients with RCC really have a higher risk of becoming infected with COVID-19 or not.

Some articles attempt to determine whether patients with cancer are at a higher risk for being infected with COVID-19 and whether they will experience greater morbidity as a result or not. We will briefly focus on two such manuscripts to illustrate how well-meaning attempts at educating the oncologic community must be interpreted with caution.

A nationwide analysis in China demonstrated that, of 1590 COVID-19 cases from 575 hospitals, 18 had a history of cancer (1 vs 0.29% of cancer incidence in the overall Chinese population, respectively), and patients with cancer were observed to have a higher risk of severe events compared with patients without cancer (39 vs 8%; p = 0.0003) ( 13 ).

Another study examined a cohort of 1,524 patients with cancer who were hospitalized from December 30, 2019 to February 13, 2020. From this group, 12 patients with cancer were identified with COVID-19 infection (0.79%), compared with 0.37% of individuals who were positive with COVID-19 in the general population of Wuhan during that same time period ( 14 ).

These studies assessing susceptibility to COVID-19 infection as well as complications directly related to infection is limited by the small number of patients with cancer in this series of heterogeneous cancer types, and the fact that hospitalized patients with cancer by definition already represent a high-risk population. Besides, without fully controlling the reasons for hospital admission or the potential confounding factors such as non-cancer comorbidities, make it difficult to draw firm conclusions about the risk of COVID-19 infection in these settings.

Articles such as these represent early attempts to assess the impact of COVID-19 on our ability to deliver high-quality cancer care, but these reports are not definitive because of some of the aforementioned limitations. The oncology community is trying to thoughtfully balance fear of COVID-19 against the direct consequences of not treating cancer in an effective or timely manner.

Difficulties in interpreting these data have also been expressed by the Editors of the *Journal of Clinical Oncology* , recognizing that the patients with cancer may indeed be at higher risk for COVID-19 infection and subsequently may experience increased morbidity and mortality compared with similar patients without cancer ( 15 ). They also acknowledge that assessing COVID-19 risk is almost certainly more complex than simply having a cancer diagnosis *per se* , and that specific cancers and therapeutic modalities may place some patients at higher risk than others.

The second question that arises from these thoughts is whether systemic renal cancer treatment with tyrosine kinase inhibitors (TKI) or immune checkpoint inhibitors (ICI) increases the risk of infection or worsens the COVID 19. Without considering the risk of exposure to infection from the treatment in itself, or for attending a health center to get this medication against the recommended social distancing; this type of treatment requires our close attention given the potential consequences of the treatment per se in a yet unknown scenario which has more questions than answers.

We can also see here that the limited cancer patient population described in the first report ( 13 ), was curiously characterized by the lack of individuals receiving anticancer immunotherapy. Indeed, only chemotherapy and surgery were cited among treatments received by patients in the month prior to developing COVID-19. Maybe, this could simply be due to the casualty of a small sample, or otherwise, it could suggest that cancer patients receiving immunotherapy are less prone to develop COVID-19 or to be admitted in hospital due to severe coronavirus symptoms. Cancer patients undergoing treatment with anti-PD-1/PD-L1 or anti-CTLA-4 immune checkpoint inhibitors (ICI) currently, constitute a growing population. Their specific susceptibility to bacterial or viral infections has not been investigated. Considering that immunotherapy with ICI is able to restore the cellular immunocompetence, as we previously suggested in the context of influenza infection, the patient undergoing immune checkpoint blockade could be more immunocompetent than cancer patients undergoing chemotherapy ( 16 , 17 ).

There are essentially two main concerns about the utilization of ICI during the COVID-19 outbreak. The first seems to be represented by the potential overlap between the coronavirus-related interstitial pneumonia and the possible pneumological toxicity from anti-PD-1/PD-L1 agents. Even if lung toxicity is not the most frequent adverse event of ICI, it can be life threatening. The overall incidence rate of ICI-related pneumonitis ranges from 2.5–5% with anti-PD-1/PD-L1 monotherapy to 7–10% with anti-CTLA-4/anti-PD-1 combination therapy ( 18 ).

The synergy between the two lung injuries, despite only being hypothetical, cannot be surely ruled out. Nevertheless, such an epidemiological coincidence should not prevent the oncologist from offering a potentially effective and often well-tolerated treatment even in the middle of the COVID-19 outbreak, since the duration of the pandemic is still currently unpredictable. This is true in particular considering the potentially curative aim of ICI treatment in the context of highly responsive diseases, such as melanoma and RCC and in the adjuvant setting even more than in the advanced disease.

Considering that underlying lung disease, particularly including interstitial pneumopathy, is considered a risk factor for ICI-related pneumonitis, it could be reasonable taking into account the risk of treating patients while they are developing an initial form of COVID-19.

The second concern seems to be represented by a possible negative interference of ICI in the pathogenesis of COVID-19. Cytokine-release syndrome (CRS) is a phenomenon of immune hyperactivation typically described in the setting of T cell-engaging immunotherapy, including CAR-T cell therapy but also anti-PD-1 agents ( 19 ). Considering these aspects, the hypothesis of a synergy between ICI mechanisms and COVID-19 pathogenesis, both contributing to a counter-producing immune hyperactivation, cannot be excluded.

Despite this, we should remember that ICI-induced CRS is a quite rare phenomenon just as that the cytokine storm is not an early event in the COVID-19 pathogenesis, indeed characterizing the late phase of its most severe manifestation, occurring in a minority of patients. It is not likely that cancer patients are still receiving ICI during this phase of the viral illness, we should be focused in delaying treatment for those patients presenting flu-like symptoms at the time of the intended ICI treatment.

Finally, according to the American Society of Clinical Oncology ASCO ( 20 ): “At this time, there is no direct evidence to support changing or withholding chemotherapy or immunotherapy in patients with cancer. Therefore, routinely withholding critical anticancer or immunosuppressive therapy is not recommended.” No reliable evidence regarding patients with any specific histology therapy (e.g. immunotherapy, tyrosine kinase inhibitors), or subpopulation of patients with cancer (e.g. children, elderly) has been identified.

In this context there are 3 recommendations:

1. There should be a doctor-patient conversation about the balance of potential harms from delaying or interrupting your systemic cancer treatment versus the potential benefits of possibly preventing or delaying COVID-19 infection.
2. That all patients receiving treatment for advanced RCC should take extra precautions to avoid risk of exposure to COVID-19.
3. It may be appropriate to adjust to less frequent dosing intervals when different schedules are considered reasonable options and/or are approved in your jurisdiction for the patient's indication.

Unfortunately, solid scientific data are lacking to guide adjustments to standard-of-care treatment regimens. Whereas sharing and discussing the expert opinions and organization may provide an initial roadmap for proceeding, the oncological community should quickly close key knowledge gaps about the incidence, morbidity and mortality of COVID-19 specific to patients with RCC, to enable evidence-based policies during this pandemic.

## DISCUSSION

The care of RCC during the COVID 19 pandemic constitutes a challenge for urologists, oncologists, and all other health professionals evolved. Nowadays there is a reduction of material resources, personal protective equipment, and there is some uncertainty because there is no available specific tests for SARs-CoV-2 for all patients, and when it is available, there are false positives and negatives results, being not possible be totally sure if the asymptomatic patient is really infected or not. Additionally, our older staffs or virus-infected colleagues may require be unavailable by several days, and the younger ones may have to be displaced from their original teams to reinforce frontline pandemic care. As a result, the urology and oncology teams may be reduced, or even stressed ( 3 ). Management focused both on patients with CRC and the scarcity of material and human resources is essential, ensuring a safe result for patients without overloading the care system.

As many RCC patients present competing risks for infection and complications of COVID 19, as age >60 years, arterial hypertension, diabetes, overweight, obesity, and smoking, it seems rational to avoid as possible, invasive treatments and repeat hospital visits, that could potentially put them under risk to be exposed to the virus during their treatments. However, there are many kidney cancer patients with aggressive locally advanced tumors, or metastatic cases, who deserve immediate surgical or systemic therapy.

In face of these dilemmas, we must take into account the natural history of heterogeneous clinical presentations of distinct stages of RCC by one side, and by the other side we must act based on the best practices recommended for the treatment of kidney malignance. From these judicious analyses, a risk-based approach must be applied for each clinical scenario, as our proposition above (summarized in Table-1 ).

**Table1:** Summarized risk-based suggested approaches (and alternative options) for renal cell carcinoma during the COVID-19 pandemic.

Stage/clinical presentation	Suggestion (s)	Alternative(s)
cT1aN0M0 (<4.0cm) and complex renal cysts (BosniaK III/IV)	Active Surveillance and postponed SurgeryΨ	Thermal ablation ***Obs.:*** For patients refractory or unavailable for surveillance.
cT1b-T2 N0M0	SurgeryΨ	Surveillance and delayed surgeryΨ (only for selected cT1b and cT2a < 7.0 cm) ***Obs.:*** CT * ou MRI * after 90 days in recommendable) ***Obs.:*** A renal biopsy could be discussed before decision between surgery or surveillance.
≥cT3 and or N+, venous thrombus	Upfront SurgeryΨ	Individualized discussion or tumor board discussion
Low Risk Metastatic	Systemic Therapy (TKI or TKI+ICI) and postponed cytorreductionΨ	Active surveillance for selected cases
Intermediate and poor Risk Metastatic	Systemic Therapy (ICI+ICIC, or ICI+ TKI)	Alternative drugs doses or scheduling intervals between applications.
		For selected intermediate risks patients with satisfactory response after systemic therapy delayed cytoreductionΨ can be discussed.
**Special conditions**		
Local Recurrences (small asymptomatic lesion)	Surveillance	Thermal ablation
Local Recurrences (symptomatic or locally invasive lesion)	Wide surgeryΨ	Systemic Therapy and delayed postponed surgery.
		Individualized discussion or tumor board
Hereditary RCC	Follow usual guidelines (surgeryΨ if >3.0 cm, except for HLRRCC syndrome (prompt resection)	Individualized discussion or tumor board discussion

* CT-Computerized Tomography;

** MR -Magnetic Resonance

# All therapeutic decisions must be preceded by a specific informed consent and based on shared decisions. Tumor boards might support decision in difficult cases.

Minimally invasive surgeries and early hospital discharge are desirable, even possible. Health professionals must not forget in using their personal protective equipment, perform safe surgery (as for open, as for minimally invasive procedures) ( 27 ).

We reinforce, that is not possible to warranty the success of all of these suggested approaches, and the environmental conditions can in major or minor grade, prejudice the treatment adherence, patient's follow-up etc. and some cases can progress quickly. Thus, we reinforce our recommendations for the use of a proper informed consent (we did not find in literature references about informed consent for cancer treatment during the COVID 19 endemic). This discussion constitutes personal opinion of authors, since our tertiary center, A.C. Camargo Cancer Center, in Brazil, has developed one informed consent for cancer treatment during this pandemic. All therapeutic choices must be based on shared decisions.

The anecdotal cases of patients unavailable to follow our directrices, or for people refractory to postponement of their treatments, must be considered as exceptions, and they must be solved after extensive discussions regarding the risks and benefits evolved.

Fortunately, actually many new cases of RCC correspond to SRM ( 21 ). The acquired experience with studies on AS for SRM, done in elderly people, and the knowledge that the majority of SRM correspond to slow growth lesions, it seems safe to extrapolate its indication, offering AS for all age's patients during the coronavirus era and a delayed treatment. For tumors >7.0 cm or local advanced tumors (≥cT3 and or N+ M0), prompt surgery is warranted, since these cases can progress in few weeks. For patients with renal vein, or vena cava thrombus, despite some isolated cases reporting of complete responses with neoadjuvant immunotherapy ( 22 ), nowadays the best approach is to perform surgery, except in cases that the tumor and thrombus seems irresectable.

If it is not complicate to postpone the treatment of SMR, and it is also no difficult to indicate immediate surgery to ≥cT3 Any N M0 cases, there is a group of patients in which the therapeutic choice seems more problematic: cT1b, T2a N0 M0. For the majority of these patients, the surgical approach seems more adequate, but we recognize that for some selected pT1b, it is possible to postpone between 60-90 days the surgeries (perhaps an abdominal cross-sectional exam could be done after 90 days), since the growth kinetics of T1b-T2 tumors is similar to SMR ( 23 ). A percutaneous biopsy ( 24 ), as an exception, could be considered, in some of these patients: if low aggressive histology is reported, the treatment might be postponed. In summary, individualized decisions seem essential for cT1b, T2a (<7.0 cm) N0 M0 cases.

Usually local or locoregional recurrence after radical nephrectomy are managed by RN ( 25 ) in face of pandemic, patients with local recurrences, might be evaluated individually: if they present small indolent lesions, we think surveillance could be an option. Conversely, if they present a large, invasive or symptomatic lesions, they could be initially undergo systemic therapy and delayed surgery (personal opinion, evidence is lacking), however some complicated cases (intestinal obstruction, bleeding, or uncontrolled pain, for example, deserves prompt wide resection, with or without intraoperative radiotherapy). Local recurrences after partial nephrectomies can be put under AS, or undergo outpatient thermal ablation, instead prompt surgery ( 25 ).

One more dilemma is: what would be the safe trigger for intervention during this pandemic for patients with hereditary RCC? In the literature, the safe trigger for intervention is the lesion size > 3.0 cm (except for HLRCC, which require prompt excision), we do not know if these patients could wait until the same trigger above suggested for sporadic SMR (4.0 cm), with an increased risk of metastatic dissemination; in this way, we recommend to adopt 3.0 cm, and an percutaneous outpatient ablative procedure could be used, instead partial nephrectomy ( 26 ).

At the same time that we suggest the use of as minimally invasive surgeries whenever possible, we reiterate that rigorous extra attention should be given to avoid the spreading SARS-CoV-2 through aerosols, that can occurs: during the installation and evacuation of pneumoperitoneum, the use of harmonic scalpels, trocars remove, specimen extraction in laparoscopic and robotic surgeries, or the use of electric scalpel in open surgeries ( 27 ). Dedicated surgical rooms and personal protective equipment are absolutely indispensable.

Upfront cytoreduction and/or resection of metastasis for patients with metastatic RCC must be discouraged. We remember that metastatic patients configure a heterogeneous group of patients. For sure, patients with intermediate or poor risks have not benefits with initial surgical approach ( 9 ), the use of immunotherapy associated or not with target therapy are largely used ( 28 - 30 ), and must not be postponed for these patients, independently during the SARS-CoV 2 pandemic ( 29 ), Salgia et al., ( 31 ) suggests the cabozantinib could replace ICI for situations of resource limitations for ICI and for patients with contra-indications (auto immune diseases) for ICI. If we are afraid in using ICI due its potential risks of pneumotoxicity, and CRS, for poor and intermediate risks individuals with RCC during this viral crisis, we could consider to extrapolate the use of this multitarget drug as an option.

Meanwhile, low risk metastatic patients (and some selected intermediate risk ones) can be benefited with upfront cytoreduction; we think at this moment that we can offer two other safe strategies instead the surgery: The first, is to start systemic therapy (TKI or TKI+ICI) and if after late evaluation, they are good responders, ( 10 ) we can discuss the cytoreduction. The second option, for selected low risk group might be the AS ( 11 ), that beyond do not compromise the outcomes of the patients in an average time around 14 months, also permits the avoidance of side effects of TKY ICI tyrosine-kinase inhibitors or immune check point inhibitors, for patients under risk of COVID-19.

Although it is desirable to reduce the need of hospital visits to get medications and to the side effects of TKI and ICI in this potentially frail population, patients with cancer and COVID 19, present worse outcomes than non-infected oncologic ones ( 13 , 14 ). Extra concern resides on severe pneumonitis or CRS, for intermediate and poor risk metastatic patients under treatment; however, there is no substitutive safe therapies until this moment. Additionally, there is no certainty when pandemic will finish, being temerarious to interrupt or postpone the initiation of systemic therapies for these group of patients, in face of the high risk of progression. Alternative dose scheduling may be discussed, an extra-caution against the viral infection during treatment must be strongly reinforced for patients and they caregivers ( 20 ).

Briefly, new studies can clarify the immune repercussions of the SARS-CoV 2 infection concomitant to ICI use: could the immune system become more efficient against the virus or the adverse effects of hyperimmune response could be potentialized?

Although we discussed usual, no usual and exceptions clinical scenarios of RCC in this manuscript, this study presents limitations. It was based on scarce literature regarding RCC and COVID 19 and it is necessary to adapt best usual recommendations for RCC for this pandemic season. Probably, this proposed risk-based recommendations, may be in some grade, influenced be authors' personal biases. For cases not contemplated in this text, for cases of difficult decisions, individualized discussions or tumor board discussion might offer the best approach to be followed.

## CONCLUSIONS

During pandemic COVID -19, a tailored stage by stage risk-based approach must be used for a safe management of RCC, aiming to not compromise the oncological outcomes of the patients. Reducing the number of invasive procedures as surgery for indolent and organ-coffined tumors, can minimize risks for RCC population, which due to its characteristics, is usually under risk of infection and complications of COVID 19, and can minimize expositional risks for urologic and oncologic teams, also. On the other hand, patients with aggressive kidney cancer deserve prompt surgical or systemic approach, despite the coronavirus virus risks. There is not enough evidence to avoid systemic therapy for metastatic RCC at this moment. All therapeutic decisions must be preceded by a specific informed consent and based on shared decisions. Tumor boards might support decision in difficult cases. Health professionals must not forget to use their personal protective equipment, perform safe surgery (as for open, as for minimally invasive procedures). More information regarding toxicities of immunotherapy and of target therapy and their implications in this scenario are waited.
